# Adapting the SPOTLIGHT Virtual Audit Tool to assess food and activity environments relevant for adolescents: a validity and reliability study

**DOI:** 10.1186/s12942-021-00258-0

**Published:** 2021-01-18

**Authors:** Oddbjørn Klomsten Andersen, Siobhan A. O’Halloran, Elin Kolle, Nanna Lien, Jeroen Lakerveld, Onyebuchi A. Arah, Mekdes K. Gebremariam

**Affiliations:** 1 Norwegian School of Sport Sciences, Ullevaal Stadion, PO Box 4014, 0806 Oslo, Norway; 2 University of Oslo, Oslo, Norway; 3 Department of Epidemiology and Data Science, Amsterdam Public Health Research Institute, Amsterdam UMC, Vrije Universiteit Amsterdam, Amsterdam, The Netherlands; 4 Upstream Team, Amsterdam UMC, Vrije Universiteit Amsterdam, Amsterdam, The Netherlands; 5 Department of Epidemiology, Fielding School of Public Health, University of California, Los Angeles (UCLA), Los Angeles, CA USA; 6 Department of Statistics, College of Letters and Science, UCLA, Los Angeles, CA USA; 7 Department of Public Health, Aarhus University, Aarhus, Denmark

**Keywords:** Built environment, Physical activity, Dietary behavior, Adolescence, Virtual audit

## Abstract

**Background:**

Physical inactivity and unhealthy diet are key behavioral determinants underlying obesity. The neighborhood environment represents an important arena for modifying these behaviors, and hence reliable and valid tools to measure it are needed. Most existing virtual audit tools have been designed to assess either food or activity environments deemed relevant for adults. Thus, there is a need for a tool that combines the assessment of food and activity environments, and which focuses on aspects of the environment relevant for youth.

**Objective:**

The aims of the present study were: (a) to adapt the SPOTLIGHT Virtual Audit Tool (S-VAT) developed to assess characteristics of the built environment deemed relevant for adults for use in an adolescent population, (b) to assess the tool’s inter- and intra-rater reliability, and (c) to assess its criterion validity by comparing the virtual audit to a field audit.

**Methods:**

The tool adaptation was based on literature review and on results of a qualitative survey investigating how adolescents perceived the influence of the environment on dietary and physical activity behaviors. Sixty streets (148 street segments) in six neighborhoods were randomly selected as the study sample. Two raters assessed the inter- and intra-rater reliability and criterion validity, comparing the virtual audit tool to a field audit. The results were presented as percentage agreement and Cohen’s kappa (κ).

**Results:**

Intra-rater agreement was found to be moderate to almost perfect (κ = 0.44–0.96) in all categories, except in the category *aesthetics* (κ = 0.40). Inter-rater agreement between auditors ranged from fair to substantial for all categories (κ = 0.24–0.80). Criterion validity was found to be moderate to almost perfect (κ = 0.56–0.82) for most categories, except *aesthetics* and *grocery stores* (κ = 0.26–0.35).

**Conclusion:**

The adapted version of the S-VAT can be used to provide reliable and valid data on built environment characteristics deemed relevant for physical activity and dietary behavior among adolescents.

## Introduction

The global burden of obesity amongst adolescents has increased more than eightfold from 0.7 to 5.6% in girls and 0.9% to 7.8% in boys, in the past 40 years [1]. Physical inactivity and unhealthy diet are key behavioral determinants underlying obesity [2]. According to ecological frameworks [3, 4], these behaviors are driven by multiple levels of influence, including built environmental factors. Broadly, the built environment can be defined as man-made structures such as neighborhoods, roads, buildings, food sources and recreational facilities in which people live, work, are educated, eat and play [5]. These characteristics are thought to facilitate or impede physical activity (PA) and healthy eating behavior [6–8]. However, very few aspects of the built environment have been consistently associated with these health behaviors among adolescents [9, 10]. Only access to school facilities/resources and access to neighborhood playgrounds and recreational facilities have been found to be associated with PA among adolescents across studies [9, 11]. There are currently no environmental factors considered to be consistently associated with dietary behavior among adolescents [10, 12–14]. The key reasons for these inconsistencies include poor quality of measurement tools, and inconsistencies in the measurement approaches used [11, 13, 15].

In public health research, field audits have traditionally been considered the criterion measurement methodology. A field audit is conducted by systematically walking the streets or parks of a given neighborhood to assess the specific characteristics with an audit tool, often in form of a checklist [16]. The main advantage of field audits is that they can provide highly detailed information about certain aspects of the built environment such as the quality and maintenance of recreational facilities and landscapes, which are rarely incorporated in secondhand data sources, such as Geographical Information Systems (GIS) [16]. However, field audits can be costly and time-consuming as they require the auditor to be physically present at the site [16]. Furthermore, concerns related to traffic and crime can make field audits impractical and unsafe in certain areas [17]. Given these concerns, researchers have opted for the use of virtual street audits.

In a virtual audit, the auditor or researcher make use of online cartographic platforms that also provides 360° photo views, such as Bing Maps or Google Street View (GSV) to walk through a neighborhood virtually [18]. Due to its vast coverage, GSV is usually the preferred platform [19]. Compared to a field audit, a virtual audit is thought to be less costly, less time-consuming and safer since the audit is performed from a personal computer [18]. Furthermore, it allows researchers to audit remote locations without being physically present at the site. Since the images are from a fixed point in time it enables the researcher to go back and validate the audits. However, there are some GSV challenges related to the limited image coverage in rural areas and the temporal validity of the images, which can make it difficult to measure the dynamic features of the built environment, such as aesthetics [20]. Google is constantly improving their GSV coverage and image quality, and a recent systematic review concluded that GSV thus far has been applied successfully in health-related research [18].

Existing virtual audit tools were mostly designed to assess built environmental characteristics deemed relevant for adults in North American or European settings and usually focus on either PA or dietary behavior [18]. However, PA and dietary behaviors are complex and interrelated [21]. It is therefore recommended to combine the measurements of food and activity environments to gain better insight into the association between the built environment and obesity [22]. The SPOTLIGHT Virtual Audit Tool (S-VAT) was the first tool to combine food and PA environments [23] and was originally designed to assess obesogenic characteristics of the built environment considered relevant for adults. The tool displayed high reliability and validity when it was tested in a Dutch setting [23]. The S-VAT was developed as a part of the large EU-funded SPOTLIGHT project [24], and has been used to investigate the association between obesogenic environments and health behavior among adults in five urban regions in France, Hungary, the United Kingdom, Belgium and the Netherlands [22, 25–27].

An important finding derived from the SPOTLIGHT project was the substantial differences in the neighborhood typologies across countries, despite the authors sampling strategies to minimize heterogeneity in neighborhoods [22]. This indicates that there are substantial inter-country differences in the built environment even within Europe. The finding might be particularly relevant for Nordic countries, as the climate allows for different activities during the winter months (e.g. cross-country skiing). To meet this challenge it has been suggested that existing measurement tools should be complemented with country-specific items to capture aspects of the environment that is geographically dependent [28]. Furthermore, the characteristics of the built environment deemed most relevant for PA and dietary behavior are likely to differ across age groups [9]. While walkability and diversity in land-use seems to be most predictive of PA behavior among adults, the presence of school infrastructure/equipment and specific recreational facilitates in the neighborhood seems to be more important among adolescents [9, 29]. Thus, existing tools such as the S-VAT may only include items that reflect a limited range of exposure variability, which might not be generalizable to other settings [22, 28] or to adolescents [9, 29]. Hence, further development, adaptations, and validation seem warranted.

The aims of the present study were: (a) to adapt the S-VAT developed to assess characteristics of the built environment deemed relevant for adults for use in an adolescent population, (b) to assess the tool’s inter- and intra-reliability, and (c) to assess its criterion validity by comparing the virtual audit to a field audit. It is hypothesized that the adapted version of the S-VAT will achieve reliable and valid results, comparable to the original S-VAT tool [23].

## Methods

The tool selected for the adaptation process was the S-VAT [23]. The S-VAT utilizes GSV in Google Earth (GE) and has 42 items (two duplicates) divided in eight categories: six walking related items (e.g. presence of sidewalk), eight cycling related items (e.g. presence of bicycle lane), two public transport items (e.g. presence of bus/tram stop), nine aesthetics items (e.g. presence of litter/graffiti), three land use-mix (e.g. type of residential buildings), five grocery store items (e.g. presence of supermarket), six food outlet items (e.g. presence of fast food restaurants), and three recreational facility items (e.g. presence of outdoor recreational facilities) [23]. We chose to keep the eight categories as they represent the main components of the built environment thought to influence health behavior [11], and to include or modify a number of items depending on relevance for adolescents, aged 12–17 years old.

### Adaptation process

The adaptation of the S-VAT tool was performed in a three-step process. First, a literature review of existing field and virtual audit tools, and environmental correlates and determinants of PA and dietary behavior was conducted to identify built environment aspects specific to adolescents, aged 12–17 years old [9, 10, 23, 29–33]. Emerging topics derived from the literature review were the importance of access to school facilities and specific PA infrastructure/equipment in the neighborhood [9, 29]. In addition, the attractiveness of the recreational facilities and parks was reported to be relevant for PA behavior and public open space visitation [29]. No additional environmental determinants were found for dietary behavior [10]. Thereafter, to identify built environment aspects specific to the geographical context, results from a qualitative survey which investigated how adolescents aged 13–14 years old from Oslo perceived the influence of the environment on dietary and PA behaviors were used (submitted work). In concordance with the first step, adolescents reported that certain recreational facilities, specifically soccer fields, were important for PA behavior. In addition, the presence of a forest was reported to be an important arena for cross-country skiing, which is the second largest sport in Norway [34]. No food outlets or stores were mentioned by the adolescents outside the already existing items in the S-VAT tool. Finally, items from the original S-VAT tool were amended accordingly.

Two duplicates and the items *public bicycle renting facilities* and *wine liquor store* were removed. The reasons for removal was either because the item was repetitive, or the item was regarded as irrelevant for adolescents or for the Norwegian context. Based on the results from the literature review and the qualitative survey, we chose to add a substantial number of items in the category *recreational facilities*. This included seven common outdoor recreational facilities and three indoor recreational facilities. The quality of the outdoor recreational facilities was added in separate items. In addition, the items *school, presence of forest, youth club, bakery* were added to the tool. The items *convenience store/grocery store* and *Cafe/Bar* was split into four individual categories, and *take away restaurants was* disaggregated into *take away* (pizza, kebab, hamburger) and *take away—others* (Indian, Asian food etc.). As more than one type of *traffic calming device* and *type of residential building* can be observed in each segment, these items were separated into individual categories. A complete list of added or modified items and their scoring/rating is available in Additional file 1.

The final tool included a total of 73 items (9 walking related items, 6 cycling related items, 2 public transport items, 13 aesthetics items, 11 land-use mix items, 6 grocery store items, 7 food outlets and 19 recreational facility items). The complete list of items is available in Additional files 2 and 3. In line with Bethlehem and colleagues [23], we adapted their standard operational procedure (SOP). The SOP is an instruction manual with detailed descriptions on how to rate each individual item. It was developed to minimize heterogeneity between audits and inconsistent scoring. For more detail, see the original S-VAT study [23]. The modifications made to the original SOP are available in Additional file 1.

The first and second authors conducted the audit using the adapted tool. From this point on, they are referred to as auditor 1 and auditor 2. Auditor 1 was a Norwegian researcher with good familiarization of the study area, while auditor 2 was an Australian researcher who was less familiar with the study area. Both auditors were trained in the use of the tool. The auditor training was conducted using the adapted SOP. Prior and during the training, the auditors were in contact with the project leader (JL) of the original S-VAT study, who gave instructions on how to rate the original items. The training was conducted by assessing a total of 36 random street segments in Oslo with various residential density (RD) and socioeconomic position (SEP). In accordance with Krieger et al. [35] the term SEP is used instead of socioeconomic status (SES), as SES does not clearly distinguish between actual resources and status. Inter-rater agreement was assessed mid-way and after completion of all the street segments. Any disagreements and misunderstandings related to the tool and its concepts were solved by discussions and further refinements of the adapted SOP to maximize homogeneity between the raters.

### Selection of study area and data collection

Sixty streets in six neighborhoods (ten in each neighborhood) in Oslo, Norway with varying RD and SEP were randomly selected as the study sample. The capital city of Norway was selected as it has a high ethnic, socioeconomic and environmental variability compared to other cities/regions [36] and the food and activity environments are likely to be more diverse compared to other cities in Norway. Oslo consists of 96 administratively defined neighborhoods. We stratified the neighborhoods in six groups based on RD and neighborhood SEP to maximize exposure variability. Residential density was obtained from Statistics Norway [37] and defined as number of inhabitants per square kilometer (inhabitants/km^2^) and stratified into tertiles (high, medium, low). Socioeconomic position was obtained from the Oslo municipality website [38], defined by the percent of the population (> 16 years) with higher education (minimum bachelor’s degree) and stratified into two groups based on median education (high/low). One neighborhood from each stratum (high SEP/low RD, high SEP/medium RD, high SEP/low RD, low SEP/high RD, low SEP/medium RD, low SEP/low RD) was randomly selected. All streets in the selected neighborhoods were identified through GE and Google Maps (GM) and ten streets from each neighborhood were randomly selected as the final study sample. The number of streets selected represented ≥ 25% of all streets identified in each respective neighborhood which has previously been suggested to be sufficient to assess the neighborhood built environment exposure [39]. All randomization was conducted using an online random number generator [40].

**Fig. 1:**
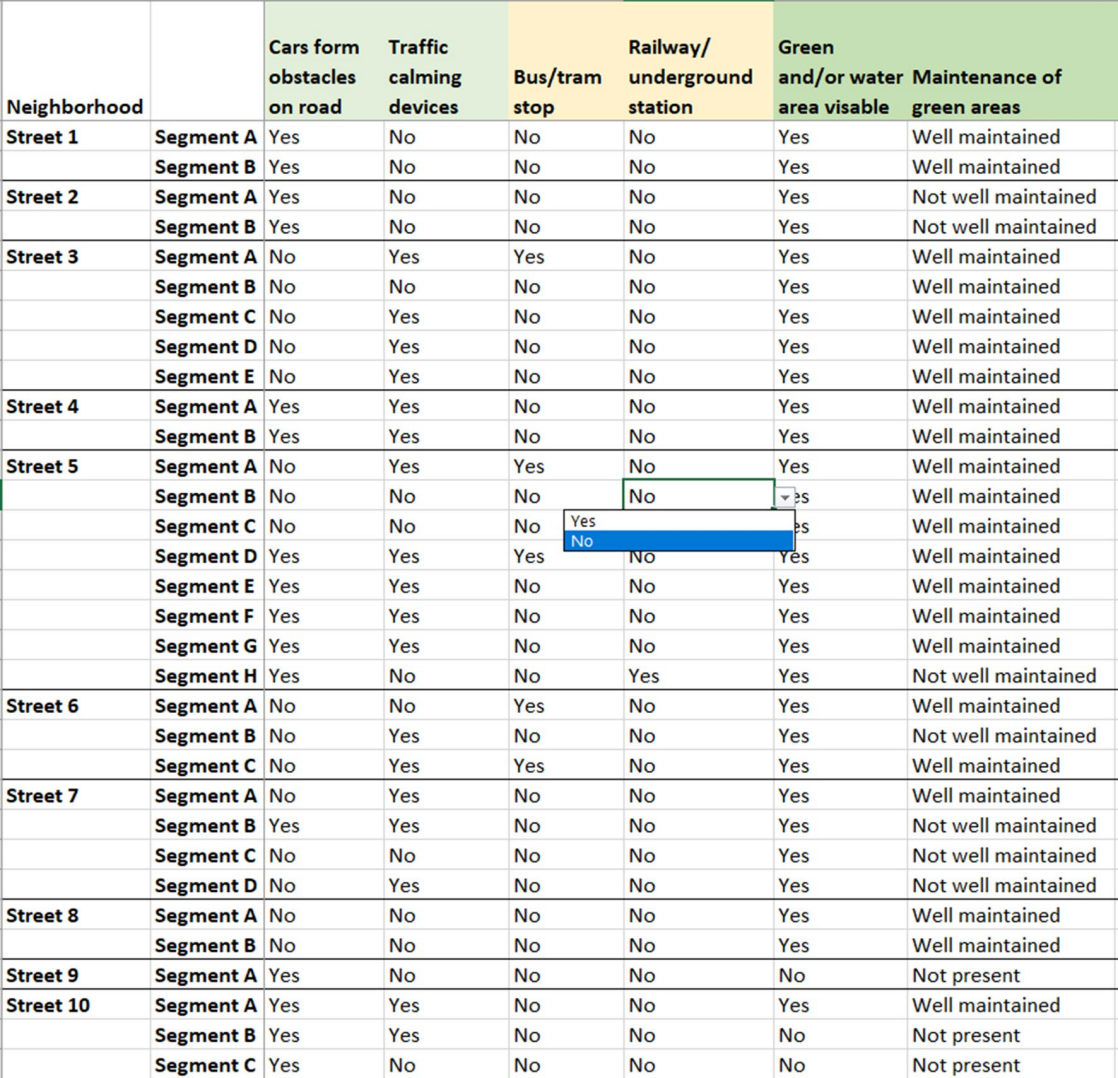
Example data extraction sheet

In line with previous studies [23, 41], the streets were further divided into street segments to ensure comparability. A street segment was defined as the length between two four-way intersections, with a minimum length of 50 m and no longer than 300 m. With the absence of a four-way intersection, an arbitrary cut-off was used at approximately 300 m. If a street crossed neighborhood boundaries, the street was assessed for the entire length, or at a maximum length of 300 m outside neighborhood borders. The streets and street segments were measured and drawn in GE using the *add path* function. This allowed for the street segments to be clearly visible in the GSV mode. Data were recorded in a Microsoft Excel spreadsheet and the data validation function was applied to mitigate typographical errors (Fig. 1). Food outlets and recreational facilities were pinpointed with x and y coordinates to enable the possibility to add them as a layer in a Geographical Information System (GIS) tool such as ArcGIS Pro. Both sides of each street segment were audited. A feature was recorded as present if it was available on minimum one side of the road (e.g. *sidewalk present*), unless otherwise stated (e.g. *sidewalk present on both side of the road*). If the quality of a feature varied across a street segment (e.g. *quality of residential gardens*), an overall quality score was given (e.g. *does residential gardens generally look trimmed and clean*). In streets with GSV images available in both driving directions, the most recent image was used to audit both sides of the road. If the timestamps were identical, both images were consulted. Times taken to complete the virtual and field audit was recorded by auditor 1. Cost analysis was conducted based on salary and expenses related to transportation (car rental, parking, fuel and bus tickets) for the field audit.

### Inter-rater reliability

The first virtual audit was conducted in the period from 9th to 16th July 2019. During the first audit, each auditor assessed 60 streets, in the six neighborhoods with varying RD and SEP independently. Inter-rater reliability was then assessed by comparing the results from the first audit of the two auditors.

### Intra-rater reliability

A second virtual audit of the same streets and street segments was conducted in the time period 30th July–6th of August 2019. Intra-rater reliability was assessed by comparing the results from the first audit to the second audit of the same 60 streets in the same six neighborhoods. To minimize recall bias, during the second audit the streets were audited in the reverse direction a minimum of 14 days after the first audit. Intra-rater reliability was performed for both auditors.

### Criterion validity

A field audit of the same streets and street segments was conducted in the time period 20th August–3rd September 2019. Criterion validity was assessed by comparing the results from the first virtual audit to the field audit. In the field audits, the auditor walked the same 60 streets in the same six neighborhoods and systematically assessing the same characteristics of interest with the use of two Apple Pro iPads®. Comparable to the virtual audit, GE was used to identify the streets and street segments, and a Microsoft Excel spread sheet with data validation was used to record the results. Both auditors conducted the field audit.

### Statistical analyses

Cohens’ kappa (κ) and weighted kappa were used to determine levels of agreement in dichotomous and categorical variables, respectively. In accordance with Landis and Koch [42], the following cut-off values were applied to determine agreement; < 0.2 poor agreement, 0.21–0.40 fair agreement, 0.41–0.60 moderate agreement, 0.61–0.80 substantial agreement, 0.81–1.00 almost perfect agreement. Landis and Koch kappa cut-offs are commonly used in built environment research [20, 23, 32, 43–45]. As Cohen’s kappa is a relative measure and a low kappa can be observed despite high agreement, we also reported percent agreement [46, 47]. Kappa values were not reported in items with zero observations. To determine homogeneity, the asymptomatic McNemar- and Stuart-Maxwell-test were performed on dichotomous and categorical variables, respectively [48–50]. An alpha value of 0.05 was used to determine statistical significance. All statistical analyses were performed with Stata/SE 16.0 (StataCorp LLC) statistical software.

## Results

A total of 181 streets were identified in the six neighborhoods. Thirty-four streets were excluded due to lack of GSV imaging. Of the remaining 147 streets, 60 streets were randomly selected and included as the final sample. The audits were completed during the summer months (July–August 2019). The GSV images were on average 5 years and 3 months old (± 3 years and 6 months) when the first virtual audit was completed (07.2019). The oldest images were from 2009 and the most recent from 2017. All the GSV images were from the months May–October. Average time taken to complete a virtual and a field audit of a neighborhood was 181 min and 40 s, and 214 min and 10 s, respectively. This included time taken to walk between streets in the field audit. The field audit was associated with an additional cost of 4818 NOK (511 USD) in salary and transportation expenses per auditor.

Due to few observations, the categories *take away (pizza, kebab, hamburger)* and *take away others (Indian, Asian *etc*.)* were merged into the category *take away restaurants* in the analysis. For similar reasons, the items *volleyball court, tennis court, basketball courts skate/BMX park, outdoor fitness facilities* and *other sports fields* were merged into the category *other outdoor facilities*. Descriptive statistics for each neighborhood are presented in Table 1. The number of observations per individual item for both auditors in each neighborhood is provided in Additional files 2 and 3.

**Table 1:** Neighborhood characteristics

Neighborhood	High socioeconomic position			Low socioeconomic position		
	HRD	MRD	LRD	HRD	MRD	LRD
RD (inhabitants/km^2^)	7140	3784	3566	18,913	4694	1443
SEP (% higher education ≥ 16 years)	61	63	65	53	29	28
Number of street segments	23	31	28	16	23	27
Avg. street segment length (m)	216 (75)	231 (71)	198 (86)	178 (59)	241 (61)	260 (62)
Distance audited (m)	4969	7388	5741	2849	5537	7029
Avg. GSV picture date (mm.yyyy)	03.2016	03.2012	08.2016	05.2016	01.2013	09.2012

*RD* residential density, *SEP* socioeconomic position, *HRD* high RD, *MRD* medium RD, *LRD* low RD, *GSV* Google Street View

### Inter-rater reliability

Mean level of agreement between auditors ranged from fair to substantial for all categories (κ = 0.24–0.80). Highest agreement was found for w*alking related items*, *public transport*, and *food outlets* (κ = 0.62–0.80) while the lowest agreement was found for *aesthetics* (κ = 0.24). Inter-rater reliability results are presented in Table 2.

**Table 2:** Inter- and intra-rater reliability and criterion validity for all items

Category	Inter-rater reliability		Intra-rater reliability Rater 1		Intra-rater reliability Rater 2		Criterion validity Rater 1		Criterion validity Rater 2	
	% Agreement	Kappa	% Agreement	Kappa	% Agreement	Kappa	% Agreement	Kappa	% Agreement	Kappa
Walking related items^a^	90	0.69	96	0.80	89	0.63	92	0.72	88	0.63
Type of street (4 categories)	84	0.69	90	0.83	89	0.77	82	0.67	86	0.70
Presence of sidewalks (yes/no)	93	0.84	99	0.97	92	0.81	97	0.92	93	0.83
Sidewalk on both sides (yes/no)	85	0.70	96	0.92	83	0.65	95	0.89	87	0.74
Pedestrian crossing available (yes/no)	86	0.72	93	0.86	87	0.73	91	0.82	87	0.72
Zebra-path (yes/no)	93	0.85	97	0.93	90	0.76	90	0.79	78	0.49
Traffic lights (yes/no)	95	0.88	97	0.93	94	0.87	95	0.89	92	0.83
Over/under pass (yes/no)	95	0.77	98	0.90	92	0.56	95	0.77	96	0.81
Presence of streetlights (yes/no)	95	0	99	0	95	− 0.01	99	0	93	− 0.03
Cars form obstacles on the road (yes/no)	87	0.74	95	0.89	77	0.54	85	0.70	81	0.60
Cycling related items^a^	95	0.48	99	0.96	95	0.47	94	0.77	92	0.50
Presence of bicycle lane (yes/no)	98	− 0.01	100	1	98	− 0.01	100	1	96	0.27
Speed limit	93	0.90	100	1	97	0.96	81	0.70	85	0.85
Type of bicycle lane (yes/no)	100	N/A	100	N/A	100	N/A	100	N/A	100	N/A
Traffic calming devices (yes/no)	78	0.55	94	0.88	75	0.47	80	0.61	68	0.38
Condition of bicycle lane (3. cat.)	100	N/A	100	N/A	100	N/A	100	N/A	100	N/A
Obstacles present bicycle lane (yes/no)	100	N/A	100	N/A	100	N/A	100	N/A	100	N/A
Public transport^a^	97	0.80	99	0.91	94	0.59	96	0.82	94	0.72
Bus/tram stop (yes/no)	94	0.81	98	0.93	90	0.68	93	0.75	91	0.74
Railway/underground station (yes/no)	99	0.79	99	0.89	97	0.49	99	0.89	97	0.70
Aesthetics^a^	81	0.24	91	0.62	87	0.40	84	0.30	82	0.26
Green/water area available (yes/no)	81	0.38	95	0.67	79	0.38	84	0.31	81	0.44
Park (yes/no)	99	− 0.01	98	0.39	96	0.55	98	0.39	95	0.40
Condition of park (3. cat.)	100	N/A	100	N/A	100	1	100	N/A	100	0
Residential gardens visible (yes/no)	90	0.74	95	0.86	84	0.58	92	0.78	87	0.62
Rating of residential gardens (well maintained/not well maintained)	90	− 0.03	99	0.80	98	− 0.01	95	− 0.02	93	− 0.02
Rating of most residential buildings (good/poor)	99	0	100	1	100	N/A	98	N/A	100	N/A
Open vacant area/parking lot available (yes/no)	73	0.46	88	0.74	77	0.52	83	0.64	75	0.49
Maintenance of green areas (well maintained/not well maintained)	45	0.06	82	0.58	80	0.26	47	− 0.15	40	0.06
Condition of sidewalks (3. cat.)	55	0.12	80	0.34	67	0.19	68	0.15	48	0.01
Presence of graffiti (yes/no)	54	0.15	78	0.55	79	0.13	64	0.29	76	0.28
Presence of litter (yes/no)	86	0.07	83	0.41	95	− 0.02	78	0.11	97	− 0.02
Presence of trees (yes/no)	88	0.21	97	0.43	85	0.23	95	0.31	89	0.23
Presence of forest (yes/no)	91	0.69	91	0.71	88	0.52	87	0.52	90	0.60
Land use-mix^a^	92	0.55	95	0.70	92	0.54	94	0.65	92	0.57
Presence of residential buildings (yes/no)	93	0.79	99	0.96	87	0.66	93	0.80	90	0.70
Detached/semidetached homes (yes/no)	90	0.80	97	0.95	92	0.83	95	0.89	89	0.77
Terraced homes (yes/no)	85	0.44	83	0.56	90	0.50	84	0.54	86	0.37
Apartment buildings ≤ 5 stories (yes/no)	81	0.61	92	0.84	78	0.55	89	0.77	80	0.60
Apartment buildings > stories (yes/no)	96	0.76	97	0.86	92	0.56	97	0.78	96	0.80
Apartment above shops (yes/no)	97	0.65	99	0.80	96	0.49	97	0.65	95	0.52
% of commercial buildings (5 categories)	83	0.29	95	0.39	83	0.46	93	0.38	83	0.47
% of industrial buildings (5 categories)	92	0.18	93	0.31	100	0.80	94	0.29	99	0.80
School (yes/no)	95	0.61	95	0.47	95	0.53	96	0.55	95	0.69
Youth clubs (yes/no)	100	N/A	100	N/A	100	N/A	100	N/A	100	N/A
Shopping mall (yes/no)	96	0.32	99	0.85	97	0	99	0.85	97	0
Grocery stores^a^	99	0.55	99	0.65	99	0.44	99	0.56	98	0.35
Supermarket (number)	99	0.96	95	0.74	98	0.77	93	0.56	92	0.43
Local food shop (number)	99	0	99	0	100	N/A	99	0.66	99	0
Bakery (number)	100	1	99	0	100	1	99	− 0.01	99	0.66
Street food market (number)	100	N/A	100	N/A	100	N/A	100	N/A	100	N/A
Small grocery store (number)	99	0	98	− 0.01	99	0	97	− 0.01	99	0
Convenience store (number)	99	0.80	99	0.66	97	− 0.01	100	1	99	0.66
Food outlets^a^	100	0.62	100	0.92	100	0.51	100	0.74	99	0.56
Restaurants (number)	99	0.57	100	1	99	0.80	99	0.80	99	0.80
Fast food outlets (number)	99	0	100	N/A	99	0	100	N/A	99	0
Take away restaurants (number)	100	0.89	100	1	100	0.75	99	0.66	97	0.59
On street vendors (number)	100	N/A	100	N/A	100	N/A	100	N/A	100	N/A
Café (number)	99	0.66	99	0.66	99	− 0.01	99	0.49	98	0.39
Bar/Pub (number)	100	1	100	1	100	1	100	1	100	1
Recreational facilities^a^	81	0.26	94	0.71	94	0.48	87	0.60	98	0.59
Playground (number)	90	0.50	91	0.66	96	0.65	92	0.65	95	0.64
Condition playground (3. cat.)	43	0	91	0.61	83	0	43	− 0.17	100	N/A
Soccer field (number)	97	0.69	98	0.71	99	0.85	95	0.59	96	0.55
Condition soccer field (3. cat.)	43	0	91	0.61	83	0	88	0.60	100	1
Other outdoor sports facilities (number)	98	0.39	99	0.89	100	0.89	95	0.66	97	0.16
Condition other sports facilities (3. cat.)	100	N/A	88	0.67	100	N/A	100	1	100	N/A
Indoor facilities (number)	98	0	99	0.85	100	N/A	99	0.85	100	N/A

*N/A* not applicable

^a^Mean category agreement

### Intra-rater reliability

Intra-rater agreement was found to be substantial to almost perfect (κ = 0.62–0.96) in all categories for auditor 1. For auditor 2 this was moderate to substantial for all categories (κ = 0.44–0.63), except *aesthetics* where fair agreement was found (κ = 0.40). Intra-rater reliability results for both auditors are presented in Table 2.

### Criterion validity

Moderate to almost perfect agreement (κ = 0.56–0.82) was found for all categories except *aesthetics* (κ = 0.30) by auditor 1. Auditor 2 found moderate to substantial agreement for all categories (κ = 0.50–0.72), except *aesthetics* and *grocery stores* (κ = 0.26–0.35). Criterion validity results for both auditors are presented in Table 2.

### Test for homogeneity

Tests for homogeneity were conducted for inter-reliability and criterion validity. Auditor 1 was more likely to rate *condition of sidewalks* as good (p ≤ 0.05), and more likely to observe *green and water areas*, *graffiti* and *trees*, compared to auditor 2 (p ≤ 0.05). Finally, higher percentage of *commercial and industrial buildings* were more likely to be observed by auditor 2 (p ≤ 0.05). Regarding criterion validity, both auditors were more likely to report poorer *quality of sidewalk* in the field audit compared to the virtual audit (p ≤ 0.05). Furthermore, both auditors reported more *traffic calming devices* in the field audit compared to the virtual audit (p ≤ 0.05). Finally, *more apartment buildings* > 5 floors were observed in the virtual audit compared to the field audit (p ≤ 0.05) by both auditors.

## Discussion

The main aims of the study were to adapt the S-VAT to assess the food and activity environments relevant for adolescents and to investigate the reliability and validity of the tool. In accordance with the hypothesis, the adapted S-VAT was found to provide reliable and valid data for most built environmental characteristics deemed relevant for PA and dietary behaviors in adolescents. The results are in agreement with previous research suggesting that virtual audit tools generally have adequate reliability and validity and are useful for assessing neighborhood characteristics [17, 18, 51–54]. Slightly lower levels of agreement were observed by auditor 2. Limited exposure variability was observed for the presence of *cycling related items*, *grocery stores, food outlets and recreational facilities*, which could explain the variability in kappa values despite almost perfect percent agreement in most items in these categories. Finally, low criterion validity was observed for *aesthetics* by both auditors indicating that the tool might be less suitable to assess this feature.

Compared to the original S-VAT, the most substantial modifications were made in the category *recreational facilities*. The decision to make these modifications were informed by existing literature and the qualitative study conducted prior to the adaptation process (submitted work). The literature suggests that certain types of recreational facilities and adjacent quality, might be of particular importance for PA behavior among adolescents [9, 29]. As the original tool only included two general items for recreational facilities (e.g. presence of *indoor recreational facility* and *presence of outdoor recreational facilities*), it was considered relevant and important to separate the more specific components*.* Determining which key recreational facilities are thought to be relevant for PA behavior among adolescents can potentially lead to the development of targeted interventions in the built environment [55].

Consistently high reliability and validity was found for the categories *walking related items* and *public transport*. This is in accordance with previous research [23, 56]. Most items were of a dichotomous nature (e.g. present/not present) and items such as *type of road*, *presence of sidewalks* and *pedestrian crossing* are clearly visible and can easily be identified in GSV. Thus, little subjective judgement is likely to occur, and high levels of agreement is expected. Some authors have pointed to the difficulties of identifying sidewalk characteristics due to parked cars blocking the view from the GSV images [57, 58]. However, this was not a major issue in the present study, possibly because the adapted S-VAT does not assess sidewalk width and curb type. Importantly, the *walking related items* and the *public transport items* are likely to be relatively stable over time. This could explain the high criterion validity despite the relatively large temporal discrepancy between when the GSV images were taken and when the field audit was carried out. However, it should be mentioned that both auditors observed significantly more *traffic calming devices* in the field audit compared to the virtual audit. This is probably due to a real difference between the photos and the field audit due to new policies in Oslo.

The lowest overall agreement for any category was observed for *aesthetics*. This finding is not surprising and has been reported by several other researchers [20, 23, 51, 52, 59, 60]. There are multiple issues related to the assessment of *aesthetics* in GSV. Many of the items in the *aesthetics* category are subjected to smaller nuances and subjective judgment [47, 61, 62]*.* Auditors can have different perceptions of what constitutes “good”, “fair” and “poor” quality, leading to systematic differences in ratings. However, the level of subjective judgment in this study was mitigated by utilizing an adapted version of the SOP used by Bethlehem et al. [23]. Nevertheless, some systematic differences between auditors did occur. For instance, *graffiti* was more frequently rated as present by auditor 1 than auditor 2, which suggests that the SOP was unsuccessfully standardized for all items.

Other issues are more directly related to GSV as a measurement tool. For instance, *condition of sidewalk* was more likely to be rated as poor in the field audit, compared to the virtual audit by both auditors which could be attributed to annual decay or poor image quality. Furthermore, the temporal validity of the images, the fixed point of the camera and poor image quality can make it challenging to assess items such as litter and graffiti [61]. The GSV images in the present study were on average five years old, which most likely affected the temporal validity of the images, resulting in poor criterion validity for both auditors. Given the potential, inherent within GSV, to derive highly detailed information about aesthetics, it is unfortunate that the adapted version of the S-VAT tool and similar tools show poor reliability and validity for many items in this category [19, 20, 59].

The categories *cycling related items*, *grocery stores, food outlets* and *recreational facilities* displayed high percentage agreement, but inconstant kappa values. High percentage agreement despite low kappa values can be observed when there is limited exposure variability. This paradox is referred to as the prevalence problem and has been observed in these categories in previous studies [20, 56]. It is attributed to the different mathematical properties of the statistical tests. While percent agreement is an absolute measure of agreement, the kappa statistics is a relative measure which also accounts for chance agreement. High agreement is expected by chance when there is low variability in the observations. By contrast, lower agreement is expected by chance when there is high variability in the observations [47]. Thus, a discordant pair has a more punitive effect on the kappa value when the variability is low. To bypass this issue, some researchers have reported the prevalence and biased adjusted Kappa (PABAK) [58, 59, 63–65]. However, the PABAK has been criticized as it tends to give disproportionate high Kappa values [66]. It has been proposed that in cases where the prevalence problem occurs and the auditors are well trained and items are of a dichotomous nature, more trust can be placed on percent agreement [47]. Nevertheless, the results in these categories should be interpreted with some caution.

A larger sample of streets could potentially have allowed to determine the actual agreement in the categories *cycling related items*, *grocery stores, food outlets* and *recreational facilities* more accurately. However, the adapted version of the S-VAT is a community tool designed to assess a vast range of exposures in residential neighborhoods. Thus, it is reasonable to assume that certain items in these categories indeed occur less frequently in the environment. This finding is supported by the results from the original S-VAT validity and reliability study, that also found low exposure variability in these categories [23]. However, low exposure variability in *cycling related items* was not observed in that study. This finding could be due to cultural differences between Norway and the Netherlands. For example, the Netherlands is especially known for its bicycle infrastructure and cycling popularity [67]. Whereas in Norway, cycling is gradually becoming more common, together with the Norwegian government committing to greater funding to support the use of bicycles, recreationally and as a mode of transport [68]. Nevertheless, when present, these features can have an important impact on adolescents’ PA and dietary behaviors [69–71]. Hence, it was considered as an important and appropriate addition to the existing tool.

Compared to intra-rater reliability, lower levels of agreement were observed for inter-rater agreement. This may be because auditor 2 was non-Norwegian and less familiar with the study area, which could indicate that there are some cultural barriers to achieving high levels of agreement. For instance, there was observed systematic differences in the ratings of quality of sidewalk between raters which could reflect differences in sidewalk quality between Australia and Norway. Furthermore, slightly lower intra-rater agreement was observed in certain items for auditor 2 compared to auditor 1. Language barriers might partially explain this issue, especially when assessing the food and activity environment, as the purpose of a building might not be obvious without being able to translate the language written on the building, leading to more guesswork. Unfortunately, as the auditors did not have any contact during the virtual audits, this was not examined thoroughly. However, discussions after the audits, revealed issues that related to understanding the Norwegian text on certain indoor recreational facilities. Notably, one of the major advantages of the virtual audit tools is the possibility to audit remote locations, thus it is important to be aware of these language translation issues.

Previous research has suggested that differences in seasonal climate can influence levels of agreement [61]. This is an important issue to address in countries with large variation in climate between the summer and winter. All the GSV images had timestamps from May–October, when the climate in Norway is quite consistent during the warmer months. However, Google has recently updated its GSV images in certain streets included in the present study and although this should theoretically increase criterion validity, some of these images were taken during snowy weather conditions in the winter. This can have major implications on criterion validity as it makes it impossible to assess certain aspects of the environment such as sidewalk characteristics, litter, quality of residential gardens, surface of sports fields, etc. Fortunately, Google has created the option of viewing older images from the same location. However, this increases the temporal discrepancy between the images obtained from GSV and the field audit which can lead to an invalid representation of the actual environment. Nevertheless, Google reports when and where they are going next, which can give researchers some predictability when planning a study in these regions [72].

## Strength and weaknesses

The present study included a randomly selected and diverse sample of neighborhoods stratified by SEP and RD, which is likely to be representative of neighborhoods in Oslo. Further validation studies in more rural areas should shed light on the generalizability of the tool’s assessments. The qualitative work conducted prior to the tool development ensured that items that are culturally and geographically specific were included. Most modifications made to the S-VAT tool were informed by international studies on environmental correlates and determinants of PA and dietary behavior deemed relevant for adolescents [9, 10, 23, 29–33]. Thus, it is reasonable to assume that the tool is generalizable to cities outside of Norway. Nevertheless, it is recommended that researchers use the present tool in combination with items that are specific for the study region in question [28]. The inclusion of auditors with different nationalities and different degree of familiarization with the study area can be considered a strength as it highlights both the tools robustness and weakness to cultural and language barriers. All reliability and validity assessments were rigorously conducted by both auditors, which further strengthen our results.

The present study has some weaknesses that should be considered. Thirty-four streets in the selected neighborhoods lacked GSV images and were thus not eligible for inclusion. These were mostly private streets leading up to private residences or very small public streets in residential areas where cars are prohibited from driving. While private streets are unlikely to influence walking and cycling behavior of adolescents in general, the small streets in residential areas that were inaccessible by the Google car can be particularly appealing for walking and cycling behavior.

The results indicate that the SOP was insufficiently standardized for certain items in the *aesthetics* category. It is likely that this contributed to poor inter-rater agreement in this category. However, auditor 1 had substantial intra-rater agreement and still displayed poor criterion validity. Thus, the poor criterion validity in the *aesthetics* category is most likely attributable to the temporal validity of the images and not to unsuccessful standardization of the SOP. This is a common problem reported in most GSV tools [18, 19]. The images in the present study were on average more than 5 years old, and certain images from smaller roads and more rural areas were 10 years old. Previous research has indicated that non-arterial streets are more likely to lack photos or have outdated images, compared to more urban areas [19, 73]. However, this problem could be partially mitigated since rural environments are thought to be more consistent than urban environments [51]. Indeed, the auditor most familiar with the study area achieved moderate to perfect criterion validity for all categories except *aesthetics*. This suggests that there was no significant change in most of the assessed environmental characteristics over time. Thus, the tool appears to be robust when it comes to tackling temporal variability.

## Conclusion

Substantial modifications were made to the original S-VAT to make it suitable to measure the characteristics of the built environment deemed relevant for adolescents. The most significant alterations were made in the category *recreational facilities*. The adapted version of the S-VAT can be used to provide reliable and valid data on most neighborhood characteristics deemed relevant for PA and dietary behavior among adolescents. It is recommended that future studies use the present tool in combination with items that are specific for the study region in question. Further validation studies in more rural areas should shed light on the generalizability of the tool’s assessments.

## Supplementary Information

**Additional file 1: Table S1.** Modified or added items to the original S-VAT tool and how to rate them.

**Additional file 2: Table S2.** Prevalence (%) of all items, across different neighborhood types (based on first auditors result).

**Additional file 3: Table S3.** Prevalence (%) of all items, across different neighborhood types (based on second auditors result).

## Data Availability

The datasets used and analyzed during the current study are available from the corresponding author on reasonable request.

## Abbreviations

GE: Google Earth
GIS: Geographical Information System
GM: Google Maps
GSV: Google Street View
PA: Physical activity
PABAK: Prevalence and bias adjusted Kappa
RD: Residential density
SEP: Socioeconomic position
SES: Socioeconomic status
SOP: Standard operational procedure
S-VAT: SPOTLIGHT Virtual Audit Tool
