# Supplementary material for: Adapting the SPOTLIGHT Virtual Audit Tool to assess food and activity environments relevant for adolescents: a validity and reliability study
Source: Int J Health Geogr. 2021 Jan 18;20:4. doi: 10.1186/s12942-021-00258-0 (PMC7814470; doi:10.1186/s12942-021-00258-0)
Supplement: Supplementary file 1 — Additional file 1: Table S1. Modified or added items to the original S-VAT tool and how to rate them. [file 12942_2021_258_MOESM1_ESM.doc]

**Additional file 1: Table S1**. Prevalence (%) of all items, across different neighbourhood types (based on second auditors result).

| **Category** | **HSEP/**  **HRD**  **N = 23** | **HSEP/ MRD**  **N = 31** | **HSEP/ LRD**  **N = 28**** | **LSEP/**  **HRD**  **N = 16** | **LSEP/**  **MRD**  **N = 23** | **LSEP/ LRD**  **N = 27** | **Total**  **N = 148**** |
| --- | --- | --- | --- | --- | --- | --- | --- |
| **Walking related items** |  |  |  |  |  |  |  |
| Type of street:  Pedestrian friendly street (number and %)  Traffic sharing road (number and %)  Regular road (number and %)  High speed traffic road (number and %) | 0 (0 %)  14 (61 %)  9 (39 %)  0 (0 %) | 0 (0 %)  26 (84 %)  5 (16 %)  0 (0 %) | 0 (0 %)  19 (68 %)  4 (14 %)  4 (14 %) | 0 (0 %)  16 (100 %)  0 (0 %)  0 (0 %) | 2 (9 %)  14 (61 %)  6 (26 %)  1 (4 %) | 1 (4 %)  18 (67 %)  8 (30 %)  0 (0 %) | 3 (2 %)  107 (72 %)  32 (22 %)  5 (3 %) |
| Sidewalk present (% yes) | 20 (87 %) | 18 (58 %) | 19 (68 %) | 15 (94 %) | 12 (52 %) | 17 (63 %) | 101 (68 %) |
| Sidewalk on both sides (% yes) | 9 (39 %) | 5 (16 %) | 13 (42 %) | 8 (50 %) | 1 (4 %) | 4 (15 %) | 40 (27 %) |
| Pedestrian crossing (% yes) | 13 (57 %) | 10 (32 %) | 15 (54 %) | 6 (38 %) | 10 (43 %) | 6 (22 %) | 60 (41 %) |
| Pedestrian crossing (type)  Zebra-path (% yes)  Traffic lights (% yes)  Over/under pass (% yes)  Not present | 7 (30 %)  6 (26 %)  2 (9 %)  10 (43 %) | 6 (19 %)  5 (16 %)  0 (0 %)  21 (68 %) | 9 (32 %)  8 (29 %)  0 (0 %)  12 (46 %) | 5 (31 %)  1 (6 %)  2 (13 %)  10 (63 %) | 7 (30 %)  0 (0 %)  3 (13 %)  13 (57 %) | 4 (15 %)  0 (0 %)  1 (4 %)  21 (78 %) | 38 (26 %)  20 (14 %)  8 (5 %)  87 (59 %) |
| Streetlights (% yes) | 20 (87 %) | 31 (100 %) | 27 (96 %) | 16 (100 %) | 20 (87 %) | 26 (96 %) | 140 (95 %) |
| Cars form obstacles on the road | 11 (48 %) | 10 (32 %) | 18 (64 %) | 13 (81 %) | 5 (22 %) | 1 (4 %) | 58 (39 %) |
| **Cycling related items** |  |  |  |  |  |  |  |
| Bicycle lane (% yes) | 0 (0%) | 0 (0 %) | 2 (7 %) | 0 (0 %) | 0 (0 %) | 0 (0 %) | 2 (1 %) |
| Speed limit  15  20  30  40  50  60  70  80  Unclear | 0 (0 %)  0 (0 %)  8 (35 %)  3 (13 %)  4 (17 %)  0 (0 %)  0 (0 %)  0 (0 %)  8 (35 %) | 0  0  14 (45 %)  11 (35 %)  0 (0 %)  0 (0 %)  0 (0 %)  0 (0 %)  5 (16 %) | 0 (0 %)  1 (4 %)  2 (7 %)  0 (0 %)  4 (14 %)  4 (14 %)  0 (0 %)  0 (0 %)  16 (57 %) | 0  1 (6 %)  5 (31 %)  2 (13 %)  0 (0 %)  0 (0 %)  0 (0 %)  0 (0 %)  7 (44 %) | 2 (9 %)  0 (0 %)  5 (22 %)  0 (0 %)  3 (13 %)  1 (4 %)  0 (0 %)  0 (0 %)  12 (52 %) | 1 (4 %)  0 (0 %)  9 (33 %)  0 (0 %)  4 (15 %)  0 (0 %)  0 (0 %)  0 (0 %)  13 (48 %) | 3 (2 %)  2 (1 %)  43 (29 %)  16 (11 %)  15 (10 %)  5 (3 %)  0 (0 %)  0 (0 %)  61 (41 %) |
| Type of bicycle lane  On road cycle lane with markings  Separate cycle lane with buffers  Shared path with pedestrians  Not present | 0 (0 %)  0 (0 %)  0 (0 %)  23 (100 %) | 0 (0 %)  0 (0 %)  0 (0 %)  31 (100 %) | 0 (0 %)  1 (4 %)  1 (4 %)  25 (89 %) | 0 (0 %)  0 (0 %)  0 (0 %)  16 (100 %) | 0 (0 %)  0 (0 %)  0 (0 %)  23 (100 %) | 0 (0 %)  0 (0 %)  0 (0 %)  27 (100 %) | 0 (0 %)  1 (1 %)  1 (1 %)  145 (98 %) |
| Condition on bicycle lane Good Fair  Poor  Not present | 0 (0 %)  0 (0 %)  0 (0 %)  23 (100 %) | 0 (0 %)  0 (0 %)  0 (0 %)  31 (100 %) | 2 (7 %)  0 (0 %)  0 (0 %)  25 (89 %) | 0 (0 %)  0 (0 %)  0 (0 %)  16 (100 %) | 0 (0 %)  0 (0 %)  0 (0 %)  23 (100 %) | 0 (0 %)  0 (0 %)  0 (0 %)  27 (100 %) | 2 (1 %)  0 (0 %)  0 (0 %)  145 (98 %) |
| Obstacles present bicycle lane (% yes) | 0 (0%) | 0 (0 %) | 0 (0 %) | 0 (0 %) | 0 (0 %) | 0 (0 %) | 0 (0 %) |
| Traffic calming devices (% yes) | 14 (61 %) | 8 (13 %) | 14 (50 %) | 4 (25 %) | 0 (0 %) | 0 (0 %) | 40 (27 %) |
| **Public transport** |  |  |  |  |  |  |  |
| Bus/tram stop (% yes) | 6 (26 %) | 7 (23 %) | 6 (21 %) | 1 (6 %) | 4 (17 %) | 9 (33 %) | 33 (22 %) |
| Railway/underground station (% yes) | 1 (4 %) | 1 (3 %) | 2 (7 %) | 0 (0 %) | 2 (9 %) | 0 (0 %) | 6 (4 %) |
| **Aesthetics** |  |  |  |  |  |  |  |
| Green and/or water area visible (% yes) | 19 (83 %) | 25 (81 %) | 9 (32 %) | 13 (81 %) | 20 (87 %) | 24 (89 %) | 110 (74 %) |
| Maintenance of green area  Well maintained  Not well maintained  Not present | 17 (74 %)  2 (9 %)  4 (17 %) | 26 (84 %)  1 (3 %)  4 (13 %) | 6 (21 %)  3 (11 %)  18 (64 %) | 12 (75 %)  1 (6 %)  3 (19 %) | 18 (78 %)  2 (9 %)  3 (13 %) | 17 (63 %)  7 (26 %)  3 (11 %) | 96 (65 %)  16 (11 %)  35 (24 %) |
| Public park (% yes) | 3 (13 %) | 0 (0 %) | 3 (11 %) | 1 (6 %) | 0 (0 %) | 0 (0 %) | 7 (5 %) |
| Condition of park Good  Fair  Poor  Not present | 3 (13 %)  0 (0 %)  0 (0 %)  20 (87 %) | 0 (0 %)  0 (0 %)  0 (0 %)  31 (100 %) | 3 (11 %)  0 (0 %)  0 (0 %)  24 (86 %) | 0 (0 %)  0 (0 %)  1 (6 %)  15 (94 %) | 0 (0 %)  0 (0 %)  0 (0 %)  23 (100%) | 0 (0 %)  0 (0 %)  0 (0 %)  27 (100%) | 6 (4 %)  0 (0 %)  1 (1 %)  140 (95 %) |
| Trees (% yes) | 21 (91 %) | 31 (100 %) | 15 (54 %) | 15 (94 %) | 23 (100 %) | 26 (96 %) | 131 (89 %) |
| Forest (% yes) | 0 (0 %) | 3 (3 %) | 0 (0 %) | 1 (6 %) | 7 (30 %) | 14 (52 %) | 25 (17 %) |
| Residential gardens (% yes) | 16 (70 %) | 31 (100 %) | 21 (75 %) | 12 ((75 %) | 16 (70 %) | 18 (67 %) | 114 (77 %) |
| Rating of condition of residential gardens Well kept condition  Not well kept condition  Not present | 16 (70 %)  0 (0 %)  7 (30 %) | 31 (100 %)  0 (0 %)  0 (0 %) | 21 (75 %)  0 (0 %)  6 (21 %) | 11 (69 %)  0 (0 %)  4 (25 %) | 16 (70 %)  0 (0 %)  7 (30 %) | 16 (59 %)  1 (4 %)  9 (33 %) | 111 (75 %)  1 (1 %)  33 (22 %) |
| Condition of residential buildings Good  Poor  Not present | 17 (74 %)  0 (0 %)  6 (26 %) | 31 (100 %)  0 (0 %)  0 (0 %) | 20 (71 %)  0 (0 %)  7 (25 %) | 13 (81 %)  0 (0 %)  3 (19 %) | 16 (70 %)  0 (0 %)  7 (30 %) | 18 (67 %)  0 (0 %)  8 (30 %) | 115 (78 %)  0 (0 %)  31 (21 %) |
| Open vacant area/parking lot (% yes) | 14 (61 %) | 5 (16 %) | 5 (18 %) | 9 (56 %) | 13 (57 %) | 13 (48 %) | 59 (40 %) |
| Sidewalk condition  Good  Fair  Poor  Under construction  Not present | 17 (74 %)  2 (9 %)  0 (0 %)  1 (4 %)  3 (13 %) | 13 (42 %)  4 (13 %)  1 (3 %)  0 (0 %)  13 (42 %) | 14 (50 %)  3 (11 %)  1 (4 %)  1 (4 %)  8 (29 %) | 8 (50 %)  6 (38 %)  1 (6 %)  0 (0 %)  1 (6 %) | 10 (43 %)  1 (4 %)  1 (4 %)  0 (0 %)  11 (48 %) | 10 (37 %)  7 (26 %)  0 (0 %)  0 (0 %)  10 (37 %) | 72 (49 %)  23 (16 %)  4 (3 %)  2 (1 %)  46 (31 %) |
| Graffiti (% yes) | 5 (22 %) | 0 (0 %) | 2 (7 %) | 4 (25 %) | 3 (13 %) | 9 (33 %) | 23 (16 %) |
| Litter (% yes) | 0 (0 %) | 0 (0 %) | 1 (4 %) | 1 (6 %) | 0 (0 %) | 0 (0 %) | 2 (1 %) |
| **Land use-mix** |  |  |  |  |  |  |  |
| Residential buildings (% yes) | 17 (74 %) | 31 (100 %) | 21 (75 %) | 13 (81 %) | 16 (69 %) | 19 (70 %) | 117 (79 %) |
| Detached/semidetached homes (% yes) | 1 (4 %) | 30 (97 %) | 18 (64 %) | 2 (13 %) | 4 (17 %) | 12 (44 %) | 67 (45 %) |
| Terraced homes (% yes) | 0 (0 %) | 3 (10 %) | 1 (4 %) | 1 (6 %) | 2 (9 %) | 4 (15 %) | 11 (7 %) |
| Apartment buildings ≤ 5 stories (% yes) | 17 (74 %) | 7 (23 %) | 9 (32 %) | 11 (69 %) | 8 (35 %) | 3 (11 %) | 55 (37 %) |
| Apartment buildings > 5 stories (% yes) | 3 (13 %) | 1 (3 %) | 1 (4 %) | 1 (6 %) | 7 (30 %) | 0 (0 %) | 13 (9 %) |
| Apartment above shops (% yes) | 8 (9 %) | 0 (0 %) | 2 (7 %) | 2 (13 %) | 0 (0 %) | 1 (4 %) | 14 (9 %) |
| % commercial buildings  0 %  25 %  50 %  75 %  100 % | 15 (65 %)  7 (30 %)  1 (4 %) 0 (0 %)  0 (0 %) | 31 (100 %)  0 (0 %)  0 (0 %)  0 (0 %)  0 (0 %) | 19 (68 %)  5 (18 %)  2 (7 %)  1 (1 %)  0 (0 %) | 12 (75 %)  4 (25 %)  0 (0 %)  0 (0 %)  0 (0 %) | 19 (83 %)  3 (13 %)  0 (0 %)  0 (0 %)  1 (4 %) | 26 (96 %)  1 (4 %)  0 (0 %)  0 (0 %)  0 (0 %) | 122 (82 %)  20 (14 %)  3 (2 %)  1 (1 %)  1 (1 %) |
| % industrial buildings  0 %  25 %  50 %  75 %  100 % | 22 (96 %)  0 (0 %)  0 (0 %)  1 (4 %)  0 (0 %) | 31 (100 %)  0 (0 %)  0 (0 %)  0 (0 %)  0 (0 %) | 27 (96 %)  0 (0 %)  0 (0 %)  0 (0 %)  0 (0 %) | 15 (94 %)  1 (6 %)  0 (0 %)  0 (0 %)  0 (0 %) | 23 (100 %)  0 (0 %)  0 (0 %)  0 (0 %)  0 (0 %) | 27 (100 %)  0 (0 %)  0 (0 %)  0 (0 %)  0 (0 %) | 145 (98 %)  1 (1 %)  0 (0 %)  1 (1 %)  0 (0 %) |
| Shopping mall (% yes) | 0 (0 %) | 0 (0 %) | 1 (4 %) | 0 (0 %) | 0 (0 %) | 0 (0 %) | 1 (1 %) |
| Youth clubs (% yes) | 0 (0 %) | 0 (0 %) | 0 (0 %) | 0 (0 %) | 0 (0 %) | 0 (0 %) | 0 (0 %) |
| Schools (% yes) | 1 (4 %) | 1 (3 %) | 1 (4 %) | 3 (19 %) | 2 (9 %) | 3 (11 %) | 11 (7 %) |
| **Grocery stores*** |  |  |  |  |  |  |  |
| Number of supermarkets | 7 (26 %) | 0 (0 %) | 4 (7 %) | 0 (0 %) | 1 (4 %) | 2 (7 %) | 14 |
| Number of local food shops | 0 (0 %) | 0 (0 %) | 0 (0 %) | 0 (0 %) | 0 (0 %) | 0 (0 %) | 0 |
| Number of bakeries | 1 (4 %) | 0 (0 %) | 0 (0 %) | 0 (0 %) | 0 (0 %) | 0 (0 %) | 1 |
| Number of street food markets | 0 (0 %) | 0 (0 %) | 0 (0 %) | 0 (0 %) | 0 (0 %) | 0 (0 %) | 0 |
| Number of small grocery stores | 0 (0 %) | 0 (0 %) | 0 (0 %) | 0 (0 %) | 0 (0 %) | 0 (0 %) | 0 |
| Number of convenience stores | 0 (0 %) | 1 (3 %) | 1 (4 %) | 0 (0 %) | 0 (0 %) | 0 (0 %) | 2 |
| **Food outlets*** |  |  |  |  |  |  |  |
| Number of restaurants | 0 (0 %) | 0 (0 %) | 4 (7 %) | 0 (0 %) | 0 (0 %) | 0 (0 %) | 4 |
| Number of fast food outlets | 0 (0 %) | 0 (0 %) | 1 (4 %) | 0 (0 %) | 0 (0 %) | 0 (0 %) | 1 |
| Number of take away pizza/burger/kebab | 1 (4 %) | 0 (0 %) | 0 (0 %) | 0 (0 %) | 0 (0 %) | 0 (0 %) | 1 |
| Number of take away sushi/Indian | 1 (4 %) | 0 (0 %) | 2 (4 %) | 0 (0 %) | 0 (0 %) | 0 (0 %) | 3 |
| Number of street vendors | 0 (0 %) | 0 (0 %) | 0 (0 %) | 0 (0 %) | 0 (0 %) | 0 (0 %) | 0 |
| Number of cafés | 0 (0 %) | 0 (0 %) | 1 (4 %) | 0 (0 %) | 0 (0 %) | 0 (0 %) | 1 |
| Number of bar/pubs | 0 (0 %) | 0 (0 %) | 0 (0 %) | 1 (6 %) | 0 (0 %) | 0 (0 %) | 1 |
| **Recreational facilities*** |  |  |  |  |  |  |  |
| Playground (% yes) | 1 (4 %) | 1 (3 %) | 0 (0 %) | 2 (13 %) | 4 (17 %) | 2 (7 %) | 10 |
| Condition of facility  Good  Fair  Poor  Not present | 0 (0 %)  0 (0 %)  0 (0 %)  22 (96 %) | 1 (3 %)  0 (0 %)  0 (0 %)  30 (97 %) | 0 (0 %)  0 (0 %)  0 (0 %)  27 (96 %) | 2 (13 %)  0 (0 %)  0 (0 %)  14 (88 %) | 3 (13 %)  0 (0 %)  0 (0 %)  19 (83 %) | 2 (7 %)  0 (0 %)  0 (0 %)  25 (93 %) | 8  0  0  137 |
| Number of soccer fields | 3 (9 %) | 3 (10 %) | 1 (4 % | 0 (0 %) | 0 (0 %) | 1 (4 %) | 8 |
| Condition of soccer fields  Good  Fair  Poor  Not present | 0 (0 %)  1 (4 %)  0 (0 %)  21 (91 %) | 3 (10 %)  0 (0 %)  0 /0 %)  28 %) | 1 (4 %)  0 (0 %)  0 (0 %)  26 (93 %) | 0 (0 %)  0 (0 %)  0 (0 %)  16 (100 %) | 0 (0 %)  0 (0 %)  0 (0 %)  23 (100 %) | 1 (4 %)  0 (0 %)  0 (0 %)  26 (96 %) | 5  1  0  140 |
| Number of volleyball courts | 0 (0 %) | 0 (0 %) | 0 ( 0 %) | 0 (0 %) | 0 (0 %) | 0 (0 %) | 0 |
| Condition of volleyball courts  Good  Fair  Poor  Not present | 0 (0 %)  0 (0 %)  0 (0 %)  23 (100 %) | 0 (0 %)  0 (0 %)  0 (0 %)  31 (100 %) | 0 (0 %)  0 (0 %)  0 (0 %)  27 (96 %) | 0 (0 %)  0 (0 %)  0 (0 %)  16 (100 %) | 0 (0 %)  0 (0 %)  0 (0 %)  23 (100 %) | 0 (0 %)  0 (0 %)  0 (0 %)  27 (100 %) | 0  0  0  147 |
| Number of tennis courts | 1 (4 %) | 6 (3 %) | 1 (4 %) | 0 (0 %) | 0 (0 %) | 0 (0 %) | 8 |
| Condition of tennis courts  Good  Fair  Poor  Not present | 1 (4 %)  0 (0 %)  0 (0 %)  22 (96 %) | 1 (3 %)  0 (0 %)  0 (0 %)  30 (97 %) | 6 (4 %)  0 (0 %)  0 (0 %)  26 (93 %) | 0 (0 %)  0 (0 %)  0 (0 %)  16 (100 %) | 0 (0 %)  0 (0 %)  0 (0 %)  23 (100 %) | 0 (0 %)  0 (0 %)  0 (0 %)  27 (100 %) | 8  0  0  144 |
| Number of basketball courts | 0 (0 %) | 0 (0 %) | 0 (0 %) | 0 (0 %) | 0 (0 %) | 0 (0 %) | 0 |
| Condition of basketball courts  Good  Fair  Poor  Not present | 0 (0 %)  0 (0 %)  0 (0 %)  23 (100 %) | 0 (0 %)  0 (0 %)  0 (0 %)  31 (100 %) | 0 (0 %)  0 (0 %)  0 (0 %)  27 (96 %) | 0 (0 %)  0 (0 %)  0 (0 %)  0 (100 %) | 0 (0 %)  0 (0 %)  0 (0 %)  23 (100 %) | 0 (0 %)  0 (0 %)  0 (0 %)  27 (100 %) | 0  0  0  147 |
| Number of other sports fields | 0 (0 %) | 0 (0 %) | 0 (0 %) | 0 (0 %) | 0 (0 %) | 0 (0 %) | 0 |
| Condition of other sports fields  Good  Fair  Poor  Not present | 0 (0 %)  0 (0 %)  0 (0 %)  23 (100 %) | 0 (0 %)  0 (0 %)  0 (0 %)  31 (100 %) | 0 (0 %)  0 (0 %)  0 (0 %)  27 (96 %) | 0 (0 %)  0 (0 %)  0 (0 %)  0 (100 %) | 0 (0 %)  0 (0 %)  0 (0 %)  23 (100 %) | 0 (0 %)  0 (0 %)  0 (0 %)  27 (100 %) | 0  0  0  147 |
| Number of skate/BMX park/bowl | 0 (0 %) | 0 (0 %) | 1 (4 %) | 0 (0 %) | 0 (0 %) | 0 (0 %) | 1 |
| Condition of skate/BMX park/bowl  Good  Fair  Poor  Not present | 0 (0 %)  0 (0 %)  0 (0 %)  23 (100 %) | 0 (0 %)  0 (0 %)  0 (0 %)  31 (100 %) | 0 (0 %)  0 (0 %)  0 (0 %)  26 (96 %) | 0 (0 %)  0 (0 %)  0 (0 %)  16 (100 %) | 0 (0 %)  0 (0 %)  0 (0 %)  23 (100 %) | 0 (0 %)  0 (0 %)  0 (0 %)  27 (100 %) | 0  0  0  146 |
| Number of outdoor fitness facilities | 0 (0 %) | 0 (0 %) | 0 (0 %) | 0 (0 %) | 0 (0 %) | 0 (0 %) | 0 |
| Condition of outdoor fitness facilities  Good  Fair  Poor  Not present | 0 (0 %)  0 (0 %)  0 (0 %)  23 (96 %) | 0 (0 %)  0 (0 %)  0 (0 %)  31 (100 %) | 0 (0 %)  0 (0 %)  0 (0 %)  27 (96 %) | 0 (0 %)  0 (0 %)  0 (0 %)  16 (100 %) | 0 (0 %)  0 (0 %)  0 (0 %)  23 (100 %) | 0 (0 %)  0 (0 %)  0 (0 %)  27 (100 %) | 0  0  0  147 |
| Number of aquatic facilities | 0 (0 %) | 0 (0 %) | 0 (0 %) | 0 (0 %) | 0 (0 %) | 0 (0 %) | 0 |
| Number of indoor gyms | 0 (0 %) | 0 (0 %) | 0 (0 %) | 0 (0 %) | 0 (0 %) | 0 (0 %) | 0 |
| Number of other indoor PA facilities | 0 (0 %) | 0 (0 %) | 0 (0 %) | 0 (0 %) | 0 (0 %) | 0 (0 %) | 0 |

HSEP = high neighbourhood socioeconomic position, LSEP = low neighbourhood socioeconomic position, HRD = high residential density, MRD = medium residential density, LRD = low residential density, * = As multiple facilities/fields can be seen in one segment, the total number of facilities/fields observed does not necessarily reflect the % of segments with facilities present.** data from one street segment is missing.
