# Supplementary material for: Adapting the SPOTLIGHT Virtual Audit Tool to assess food and activity environments relevant for adolescents: a validity and reliability study
Source: Int J Health Geogr. 2021 Jan 18;20:4. doi: 10.1186/s12942-021-00258-0 (PMC7814470; doi:10.1186/s12942-021-00258-0)
Supplement: Supplementary file 2 — Additional file 2: Table S2. Prevalence (%) of all items, across different neighborhood types (based on first auditors result). [file 12942_2021_258_MOESM2_ESM.doc]

**Additional file 2: Table S2**. Prevalence (%) of all items, across different neighbourhood types (based on first auditors result).

| **Category** | **HSEP/**  **HRD**  **N = 23** | **HSEP/ MRD**  **N = 31** | **HSEP/ LRD**  **N = 28** | **LSEP/**  **HRD**  **N = 16** | **LSEP/**  **MRD**  **N = 23** | **LSEP/ LRD**  **N = 27** | **Total**  **N = 148** |
| --- | --- | --- | --- | --- | --- | --- | --- |
| **Walking related items** |  |  |  |  |  |  |  |
| Type of street:  Pedestrian friendly street (number and %)  Traffic sharing road (number and %)  Regular road (number and %)  High speed traffic road (number and %) | 0 (0 %)  12 (52 %)  11 (48 %)  0 (0 %) | 0 (0 %)  22 (71 %)  9 (29 %)  0 (0 %) | 0 (0 %)  16 (57 %)  8 (29 %)  4 (14 %) | 0 (0 %)  16 (100 %)  0 (0 %)  0 (0 %) | 1 (4 %)  13 (57 %)  7 (30 %)  2 (9 %) | 1 (4 %)  17 (63 %)  9 (33 %)  0 (0 %) | 2 (1 %)  96 (65 %)  44 (30 %)  6 (4 %) |
| Sidewalk present (% yes) | 20 (87 %) | 19 (61 %) | 22 (79 %) | 15 (94 %) | 11 (48 %) | 16 (59 %) | 103 (70 %) |
| Sidewalk on both sides (% yes) | 9 (39 %) | 5 (16 %) | 14 (50 %) | 10 (63 %) | 0 (0 %) | 1 (4 %) | 39 (26 %) |
| Pedestrian crossing (% yes) | 13 (57 %) | 17 (55 %) | 15 (54 %) | 7 (44 %) | 11 (48 %) | 5 (19 %) | 68 (46 %) |
| Pedestrian crossing (type)  Zebra-path (% yes)  Traffic lights (% yes)  Over/under pass (% yes)  Not present | 7 (30 %)  6 (26 %)  2 (9 %)  10 (43 %) | 10 (32 %)  9 (29 %)  0 (0 %)  14 (45 %) | 7 (25 %)  9 (32 %)  0 (0 %)  13 (46 %) | 6 (38 %)  1 (6 %)  0 (0 %)  9 (56 %) | 7 (30 %)  0 (0 %)  4 (17 %)  12 (52 %) | 3 (11 %)  0 (0 %)  2 (7 %)  22 (81 %) | 40 (27 %)  25 (17 %)  8 (5 %)  78 (53 %) |
| Streetlights (% yes) | 23 (100 %) | 31 (100 %) | 28 (100 %) | 16 (100 %) | 23 (100 %) | 27 (100 %) | 148 (100 %) |
| Cars form obstacles on the road | 11 (48 %) | 16 (52 %) | 19 (68 %) | 13 (81 %) | 5 (22 %) | 3 (11 %) | 67 (45 %) |
| **Cycling related items** |  |  |  |  |  |  |  |
| Bicycle lane (% yes) | 0 (0%) | 0 (0%) | 1 (4 %) | 0 (0 %) | 1 (4 %) | 0 (0 %) | 2 (1 %) |
| Speed limit  15  20  30  40  50  60  70  80  Unclear | 0 (0 %)  0 (0 %)  5 (22 %)  3 (13 %)  4 (17 %)  0 (0 %)  0 (0 %)  0 (0 %)  11 (48 %) | 0 (0 %)  0 (0 %)  16 (52 %)  12 (39 %)  0 (0 %)  0 (0 %)  0 (0 %)  0 (0 %)  3 (10 %) | 0 (0 %)  0 (0 %)  2 (7 %)  0 (0 %)  5 (18 %)  4 (14 %)  0 (0 %)  0 (0 %)  17 (61 %) | 0 (0 %)  0 (0 %)  5 (31 %)  2 (13 %)  0 (0 %)  0 (0 %)  0 (0 %)  0 (0 %)  9 (56 %) | 1 (4 %)  0 (0 %)  5 (22 %)  0 (0 %)  3 (13 %)  2 (9 %)  0 (0 %)  0 (0 %)  12 (52 %) | 1 (4 %)  0 (0 %)  9 (33 %)  0 (0 %)  7 (26 %)  0 (0 %)  0 (0 %)  0 (0 %)  10 (37 %) | 1 (1 %)  0 (0 %)  42 (28 %)  17 (11 %)  19 (13 %)  6 (4 %)  0 (0 %)  0 (0 %)  62 (42 %) |
| Type of bicycle lane  On road cycle lane with markings  Separate cycle lane with buffers  Shared path with pedestrians  Not present | 0 (0 %)  0 (0 %)  0 (0 %)  23 (100 %) | 0 (0 %)  0 (0%)  0 (0 %)  31 (100 %) | 0 (0 %)  1 (4 %)  0 (0 %)  27 (96 %) | 0 (0 %)  0 (0 %)  0 (0 %)  16 (100 %) | 0 (0 %)  1 (4 %)  0 (0 %)  23 (100 %) | 0 (0 %)  0 (0 %)  0 (0 %)  27 (100 %) | 0 (0 %)  2 (1 %)  0 (0 %)  146 (99 %) |
| Condition on bicycle lane Good Fair  Poor  Not present | 0 (0 %)  0 (0%)  0 (0 %)  23 (100 %) | 0 (0 %)  0 (0%)  0 (0 %)  31 (100 %) | 1 (4 %)  0 (0 %)  0 (0 %)  27 (96 %) | 0 (0 %)  0 (0 %)  0 (0 %)  16 (100 %) | 1 (4 %)  0 (0 %)  0 (0 %)  22 (96 %) | 0 (0 %)  0 (0 %)  0 (0 %)  27 (100 %) | 2 (1 %)  0 (0 %)  0 (0 %)  146 (99 %) |
| Obstacles present bicycle lane (% yes) | 0 (0%) | 0 (0%) | 0 (0%) | 0 (0%) | 0 (0%) | 0 (0%) | 0 (0%) |
| Traffic calming devices (% yes) | 14 (61 %) | 18 (58 %) | 20 (71 %) | 4 (25 %) | 7 (30 %) | 8 (30 %) | 71 (48 %) |
| **Public transport** |  |  |  |  |  |  |  |
| Bus/tram stop (% yes) | 4 (17 %) | 5 (16 %) | 5 (18 %) | 1 (6 %) | 2 (9 %) | 7 (26 %) | 24 (16 %) |
| Railway/underground station (% yes) | 1 (4 %) | 1 (3 %) | 2 (7 %) | 0 (0 %) | 0 (0 %) | 0 (0 %) | 4 (3 %) |
| **Aesthetics** |  |  |  |  |  |  |  |
| Green and/or water area visible (% yes) | 21 (91 %) | 28 (90 %) | 20 (61 %) | 15 (94 %) | 22 (96 %) | 25 (93 %) | 131 (89 %) |
| Maintenance of green area  Well maintained  Not well maintained  Not present | 15 (65 %)  6 (26 %)  2 (9 %) | 21 (68 %)  7 (23 %)  3 (10 %) | 16 (57 %)  4 (14 %)  8 (29 %) | 13 (81 %)  2 (13 %)  1 (6 %) | 12 (52 %)  9 (39 %)  1 (4 %) | 12 (44 %)  13 (48 %)  2 (7 %) | 89 (60 %)  41 (28 %)  17 (11 %) |
| Public park (% yes) | 0 (0 %) | 0 (0 %) | 0 (0 %) | 0 (0 %) | 0 (0 %) | 1 (4 %) | 1 (1 %) |
| Condition of park Good  Fair  Poor  Not present | 0 (0 %)  0 (0 %)  0 (0 %)  23 (100 %) | 0 (0 %)  0 (0 %)  0 (0 %)  31 (100 %) | 0 (0 %)  0 (0 %)  0 (0 %)  28 (100 %) | 0 (0 %)  0 (0 %)  0 (0 %)  16 (100 %) | 0 (0 %)  0 (0 %)  0 (0 %)  23 (100 %) | 1 (4 %)  0 (0 %)  0 (0 %)  26 (96 %) | 1 (1 %)  0 (0 %)  0 (0 %)  147 (99 %) |
| Trees (% yes) | 21 (91 %) | 31 (100 %) | 24 (86 %) | 16 (100 %) | 23 (100 %) | 26 (96 %) | 141 (95 %) |
| Forest (% yes) | 0 (0 %) | 2 (6 %) | 1 (4 %) | 0 (0 %) | 9 (39 %) | 14 (52 %) | 26 (18 %) |
| Residential gardens (% yes) | 15 (65 %) | 28 (90 %) | 20 (71 %) | 15 (94 %) | 16 (70 %) | 16 (59 %) | 110 (74 %) |
| Rating of condition of residential gardens Well kept condition  Not well kept condition  Not present | 14 (61 %)  6 (26 %)  3 (13 %) | 28 (90 %)  0 (0 %)  3 (10 %) | 20 (71 %)  0 (0 %)  8 (29 %) | 14 (88 %)  1 (6 %)  1 (6 %) | 16 (70 %)  0 (0 %)  7 (30 %) | 15 (56 %)  1 (4 %)  11 (41 %) | 107 (72 %)  8 (5 %)  33 (22 %) |
| Condition of residential buildings Good  Poor  Not present | 18 (78 %)  0 (0 %)  5 (22 %) | 30 (97 %)  0 (0 %)  1 (3 %) | 20 (71 %)  0 (0 %)  8 (29 %) | 14 (88 %)  1 (6 %)  1 (6 %) | 16 (70 %)  0 (0 %)  7 (30 %) | 17 (63 %)  0 (0 %)  10 (37 %) | 115 (78 %)  0 (0 %)  32 (22 %) |
| Open vacant area/parking lot (% yes) | 14 (61 %) | 5 (16 | 8 (29 %) | 7 (44 %) | 11 (48 %) | 12 (44 %) | 57 (39 %) |
| Sidewalk condition  Good  Fair  Poor  Under construction  Not present | 18 (78 %)  1 (4 %)  0 (0 %)  1 (4 %)  3 (13 %) | 16 (52 %)  3 (10 %)  0 (0 %)  0 (0 %)  12 (39 %) | 15 (54 %)  5 (18 %)  0 (0 %)  1 (4 %)  7 (25 %) | 14 (88 %)  1 (6 %)  0 (0 %)  0 (0 %)  1 (6 %) | 10 (43 %)  1 (4 %)  0 (0 %)  0 (0 %)  12 (52 %) | 15 (56 %)  1 (4 %)  0 (0 %)  0 (0 %)  11 (41 %) | 88 (59 %)  12 (8 %)  0 (0 %)  2 (1 %)  46 (31 %) |
| Graffiti (% yes) | 9 (39 %) | 18 (58 %) | 17 (61 %) | 10 (63 %) | 15 (65 %) | 15 (56 %) | 84 (57 %) |
| Litter (% yes) | 1 (4 %) | 0 (0 %) | 5 (18 %) | 2 (13 %) | 9 (39 %) | 3 (11 %) | 20 (14 %) |
| **Land use-mix** |  |  |  |  |  |  |  |
| Residential buildings (% yes) | 18 (78 %) | 30 (97 %) | 22 (79 %) | 15 (94 %) | 16 (70 %) | 17 (63 %) | 118 (80 %) |
| Detached/semidetached homes (% yes) | 4 (17 %) | 30 (97 %) | 20 (71 %) | 2 (13 %) | 5 (22 %) | 14 (52 %) | 75 (51 %) |
| Terraced homes (% yes) | 0 (0 %) | 6 (19 %) | 7 (25 %) | 1 (6 %) | 6 (26 %) | 5 (19 %) | 25 (17 %) |
| Apartment buildings ≤ 5 stories (% yes) | 18 (78 %) | 5 (16 %) | 4 (14 %) | 12 (75 %) | 7 (30 %) | 0 (0 %) | 46 (31 %) |
| Apartment buildings > 5 stories (% yes) | 4 (17 %) | 0 (0 %) | 0 (0 %) | 3 (19 %) | 5 (22 %) | 0 (0 %) | 12 (8 %) |
| Apartment above shops (% yes) | 1 (4 %) | 0 (0 %) | 1 (4 %) | 1 (6 %) | 0 (0 %) | 0 (0 %) | 2 (1 %) |
| % commercial buildings  0 %  25 %  50 %  75 %  100 % | 22 (96 %)  0 (0 %)  1 (4 %)  0 (0 %)  0 (0 %) | 30 (97 %)  1 (3 %)  0 (0 %)  0 (0 %)  0 (0 %) | 26 (93 %)  0 (0 %)  2 (7 %)  0 (0 %)  0 (0 %) | 16 (100 %)  0 (0 %)  0 (0 %)  0 (0 %)  0 (0 %) | 21 (91 %)  2 (9 %)  0 (0 %)  0 (0 %)  0 (0 %) | 27 (100 %)  0 (0 %)  0 (0 %)  0 (0 %)  0 (0 %) | 142 (96 %)  3 (2 %)  3 (2 %)  0 (0 %)  0 (0 %) |
| % industrial buildings  0 %  25 %  50 %  75 %  100 % | 18 (78 %)  0 (0 %)  1 (4 %)  0 (0 %)  4 (17 %) | 31 (100 %)  0 (0 %)  0 (0 %)  0 (0 %)  0 (0 %) | 27 (96 %)  0 (0 %)  1 (4 %)  0 (0 %)  0 (0 %) | 14 (88 %)  1 (6 %)  1 (6 %)  0 (0 %)  0 (0 %) | 22 (96 %)  0 (0 %)  0 (0 %)  1 (4 %)  0 (0 %) | 26 (96 %)  0 (0 %)  1 (4 %)  0 (0 %)  0 (0 %) | 138 (93 %)  1 (1 %)  4 (3 %)  1 (1 %)  4 (3 %) |
| Shopping mall (% yes) | 1 (4 %) | 0 (0 %) | 2 (7 %) | 1 (6 %) | 0 (0 %) | 0 (0 %) | 4 (3 %) |
| Youth clubs (% yes) | 0 (0 %) | 0 (0 %) | 0 (0 %) | 0 (0 %) | 0 (0 %) | 0 (0 %) | 0 (0 %) |
| Schools (% yes) | 0 (0 %) | 0 (0 %) | 1 (4 %) | 1 (6 %) | 2 (9 %) | 4 (15 %) | 8 (5 %) |
| **Grocery stores** |  |  |  |  |  |  |  |
| Number of supermarkets* | 7 (26 %) | 0 (0 %) | 4 (7 %) | 0 (0 %) | 1 (4 %) | 1 (4 %) | 13 |
| Number of local food shops* | 0 (0 %) | 0 (0 %) | 1 (4 %) | 0 (0 %) | 0 (0 %) | 0 (0 %) | 1 |
| Number of bakeries* | 1 (4 %) | 0 (0 %) | 0 (0 %) | 0 (0 %) | 0 (0 %) | 0 (0 %) | 1 |
| Number of street food markets* | 0 (0%) | 0 (0 %) | 0 (0 %) | 0 (0 %) | 0 (0 %) | 0 (0 %) | 0 |
| Number of small grocery stores* | 0 (0 %) | 0 (0 %) | 0 (0 %) | 1 (6 %) | 0 (0 %) | 0 (0 %) | 1 |
| Number of convenience stores* | 0 (0 %) | 1 (3 %) | 1 (4 %) | 0 (0 %) | 1 (4 %) | 0 (0 %) | 3 |
| **Food outlets** |  |  |  |  |  |  |  |
| Number of restaurants* | 0 (0 %) | 0 (0 %) | 3 (7 %) | 0 (0 %) | 0 (0 %) | 0 (0 %) | 3 |
| Number of fast food outlets* | 0 (0 %) | 0 (0 %) | 0 (0 %) | 0 (0 %) | 0 (0 %) | 0 (0 %) | 0 |
| Number of take away pizza/burger/kebab* | 1 (4 %) | 0 (0 %) | 1 (4 %) | 0 (0 %) | 0 (0 %) | 0 (0 %) | 2 |
| Number of take away sushi/Indian* | 1 (4 %) | 0 (0 %) | 3 (4 %) | 0 (0 %) | 0 (0 %) | 0 (0 %) | 4 |
| Number of street vendors* | 0 (0 %) | 0 (0 %) | 0 (0 %) | 0 (0 %) | 0 (0 %) | 0 (0 %) | 0 |
| Number of cafés* | 0 (0 %) | 0 (0 %) | 2 (7 %) | 0 (0 %) | 0 (0 %) | 0 (0 %) | 2 |
| Number of bar/pubs* | 0 (0 %) | 0 (0 %) | 0 (0 %) | 1 (6 %) | 0 (0 %) | 0 (0 %) | 1 |
| **Recreational facilities** |  |  |  |  |  |  |  |
| Playground (% yes) | 5 (22 %) | 1 (3 %) | 2 (7 %) | 6 (38 %) | 6 (26 %) | 3 (11 %) | 23 (16 %) |
| Condition of facility  Good  Fair  Poor  Not present | 2 (9 %)  1 (4 %)  0 (0 %)  18 (78 %) | 1 (3 %)  0 (0 %)  0 (0 %)  30 (97 %) | 0 (0 %)  2 (7 %)  0 (0 %)  26 (93 %) | 2 (13 %)  3 (19 %)  1 (6 %)  10 (63 %) | 0 (0 %)  6 (26 %)  0 (0 %)  17 (74 %) | 1 (4 %)  2 (7 %)  0 (0 %)  23 (85 %) | 6 (4 %)  14 (9 %)  1 (1 %)  124 (84 %) |
| Number of soccer fields** | 4 (9 %) | 5 (10 %) | 1 (4 %) | 0 (0 %) | 1 (4 %) | 1 (4 %) | 12 |
| Condition of soccer fields  Good  Fair  Poor  Not present | 4 (9 %)  0 (0 %)  0 (0 %)  21 (91 %) | 5 (10 %)  0 (0 %)  0 (0 %)  28 (90 %) | 1 (100 %)  0 (0 %)  0 (0 %)  27 (96 %) | 0 (0 %)  0 (0 %)  0 (0 %)  16 (100 %) | 0 (0 %)  1 (4 %)  0 (0 %)  22 (96 %) | 0 (0 %)  1 (4 %)  0 (0 %)  26 (96 %) | 10  2  0  140 (95 %) |
| Number of volleyball courts* | 0 (0 %) | 0 (0 %) | 0 (0 %) | 0 (0 %) | 0 (0 %) | 0 (0 %) | 0 |
| Condition of volleyball courts  Good  Fair  Poor  Not present | 0 (0 %)  0 (0 %)  0 (0 %)  23 (100 %) | 0 (0 %)  0 (0 %)  0 (0 %)  31 (100 %) | 0 (0 %)  0 (0 %)  0 (0 %)  28 (100 %) | 0 (0 %)  0 (0 %)  0 (0 %)  16 (100 %) | 0 (0 %)  0 (0 %)  0 (0 %)  23 (100 %) | 0 (0 %)  0 (0 %)  0 (0 %)  27 (100 %) | 0  0  0  148 (100 %) |
| Number of tennis courts* | 1 (4 %) | 2 (3 %) | 7 (7 %) | 0 (0 %) | 0 (0 %) | 0 (0 %) | 10 |
| Condition of tennis courts  Good  Fair  Poor  Not present | 1 (4 %)  0 (0 %)  0 (0 %)  22 (96 %) | 2 (3 %)  0 (0 %)  0 (0 %)  30 (97 %) | 7 (7 %)  0 (0 %)  0 (0 %)  26 (93 %) | 0 (0 %)  0 (0 %)  0 (0 %)  16 (100 %) | 0 (0 %)  0 (0 %)  0 (0 %)  23 (100 %) | 0 (0 %)  0 (0 %)  0 (0 %)  27 (100 %) | 10  0  0  144 (97 %) |
| Number of basketball courts* | 0 (0 %) | 0 (0 %) | 0 (0 %) | 0 (0 %) | 1 (4 %) | 1 (4 %) | 2 |
| Condition of basketball courts  Good  Fair  Poor  Not present | 0 (0 %)  0 (0 %)  0 (0 %)  23 (100 %) | 0 (0 %)  0 (0 %)  0 (0 %)  31 (100 %) | 0 (0 %)  0 (0 %)  0 (0 %)  28 (100 %) | 0 (0 %)  0 (0 %)  0 (0 %)  16 (100 %) | 0 (0 %)  1 (4 %)  0 (0 %)  22 (96 %) | 0 (0 %)  1 (4 %)  0 (0 %)  26 (96 %) | 0  2  0  146 (99 %) |
| Number of other sports fields* | 0 (0 %) | 0 (0 %) | 0 (0 %) | 0 (0 %) | 0 (0 %) | 0 (0 %) | 0 |
| Condition of other sports fields  Good  Fair  Poor  Not present | 0 (0%)  0 (0 %)  0 (0 %)  23 (100 %) | 0 (0 %)  0 (0 %)  0 (0 %)  31 (100 %) | 0 (0 %)  0 (0 %)  0 (0 %)  28 (100 %) | 0 (0 %)  0 (0 %)  0 (0 %)  16 (100 %) | 0 (0 %)  0 (0 %)  0 (0 %)  23 (100 %) | 0 (0 %)  0 (0 %)  0 (0 %)  27 (100 %) | 0  0  0  148 (100 %) |
| Number of skate/BMX park/bowl* | 0 (0 %) | 0 (0 %) | 0 (0 %) | 0 (0 %) | 0 (0 %) | 0 (0 %) | 0 |
| Condition of skate/BMX park/bowl  Good  Fair  Poor  Not present | 0 (0 %)  0 (0 %)  0 (0 %)  23 (100 %) | 0 (0 %)  0 (0 %)  0 (0 %)  31 (100 %) | 0 (0 %)  0 (0 %)  0 (0 %)  28 (100 %) | 0 (0 %)  0 (0 %)  0 (0 %)  16 (100 %) | 0 (0 %)  0 (0 %)  0 (0 %)  23 (100 %) | 0 (0 %)  0 (0 %)  0 (0 %)  27 (100 %) | 0  0  0  148 (100 %) |
| Number of outdoor fitness facilities* | 0 (0 %) | 0 (0 %) | 0 (0 %) | 0 (0 %) | 0 (0 %) | 0 (0 %) | 0 |
| Condition of outdoor fitness facilities  Good  Fair  Poor  Not present | 0 (0 %)  0 (0 %)  0 (0 %)  23 (100 %) | 0 (0 %)  0 (0 %)  0 (0 %)  31 (100 %) | 0 (0 %)  0 (0 %)  0 (0 %)  28 (100 %) | 0 (0 %)  0 (0 %)  0 (0 %)  16 (100 %) | 0 (0 %)  0 (0 %)  0 (0 %)  23 (100 %) | 0 (0 %)  0 (0 %)  0 (0 %)  27 (100 %) | 0  0  0  148 (100 %) |
| Number of aquatic facilities* | 0 (0 %) | 0 (0 %) | 0 (0 %) | 0 (0 %) | 0 (0 %) | 0 (0 %) | 0 |
| Number of indoor gyms* | 1 (4 %) | 0 (0 %) | 1 (4 %) | 0 (0 %) | 0 (0 %) | 0 (0 %) | 2 |
| Number of other indoor PA facilities* | 0 (0 %) | 1 (3 %) | 0 (0 %) | 0 (0 %) | 0 (0 %) | 0 (0 %) | 1 |

HSEP = high neighbourhood socioeconomic position, LSEP = low neighbourhood socioeconomic position, HRD = high residential density, MRD = medium residential density, LRD = low residential density, * = As multiple facilities/fields can be seen in one segment, the total number of facilities/fields observed does not necessarily reflect the % of segments with facilities present.
