# Supplementary material for: Adapting the SPOTLIGHT Virtual Audit Tool to assess food and activity environments relevant for adolescents: a validity and reliability study
Source: Int J Health Geogr. 2021 Jan 18;20:4. doi: 10.1186/s12942-021-00258-0 (PMC7814470; doi:10.1186/s12942-021-00258-0)
Supplement: Supplementary file 3 — Additional file 3: Table S3. Prevalence (%) of all items, across different neighborhood types (based on second auditors result). [file 12942_2021_258_MOESM3_ESM.doc]

**Additional file 3: Table S3.** Modified or added items to the original S-VAT tool and how to rate them.

| **Category/item** |  | **Description** |
| --- | --- | --- |
| **Walking related items** |  |  |
| Type of street: |  |  |
|  | Pedestrian friendly street | Area where vehicles are allowed to drive max. 15 km/h. Road is shared with pedestrians, cyclists and cars. Children can play on the streets. |
|  | Traffic sharing road | Road in residential area. Maximum speed can differ from 20 to 40 km/t. Roads do typically not have center line markings. |
|  | Regular road | Road in urban area where cars, cyclists and/or pedestrians have separated paths, with or without a buffer. Traffic speed does not exceed 50 km/h. |
|  | Road with high-speed traffic | Main road that connects towns and cities, where speed exceeds 50 km/h. (i.e. ring 3) |
| **Aesthetics** |  |  |
| Forest | Yes/No | Rate if visible from the road. i.e. if all you see is threes from the sidewalk – then rate it as a forest. |
| **Land use-mix** |  |  |
| Shopping mall | Yes/No and coordinates | Multiple shops housed in one building |
| Youth clubs | Yes/No and coordinates | Often called "fritidsklubb/ungdomsklubb" in Norwegian |
| School | Yes/No and coordinates |  |
| **Grocery stores** |  |  |
| Small grocery store | Number and coordinates | Smaller version of a supermarket but is from a local chain and can have different opening hours than larger supermarkets (Bunnpris, Joker, Jacobs & Matkroken). |
| Bakery | Number and coordinates | Sells bread and sweets (i.e. Baker Hansen, W.B Samson) |
| **Food outlets** |  |  |
| Take away kebab, pizza and/or Burger | Number and coordinates | ‘Ready to eat’ food defined as complete meals that need no further preparation & are bought from food outlets to either eat in, take away or be delivered. |
| Take away other | Number and coordinates | Any other ‘ready to eat’ food outlet/restaurant. ‘Ready to eat’ defined as complete meals that need no further preparation & are bought from food outlets to either eat in, take away or be delivered. E.g. sushi place or deli de luca. |
| Bar/Pub | Number and coordinates | A place that sells alcoholic beverages. |
| **Recreational facilities** |  |  |
| Playground | Yes/No and coordinates |  |
| Condition of facility | Good | Well maintained, new paint and free of rust and cracks. |
|  | Fair | Fair maintained, can have pale colors, some rust or cracks, typically older playgrounds. |
|  | Poor | Poor maintained, substantial amount of rust or cracks, not been maintained in years. |
| Soccer fields | Number and coordinates |  |
| Condition of facility | Good | Well maintained (grass cut if natural grass), lighting, even playing surface, intact goals. |
|  | Fair | Fair maintained, uneven playing surface in some areas, goals can have minor defects (small holes in nets). |
|  | Poor | Poor maintained, uneven playing surface, goals have substantial defects (missing nets etc.). |
| Volleyball fields | Number and coordinates |  |
| Condition of facility | Good | Well maintained, even playing surface (if hard court), enough sand (if beach volley), lighting, intact nets. |
|  | Fair | Fair maintained, uneven playing surface in some areas (if hard court), inadequate amounts of sand in some areas (if beach volley), minor defects on nets (i.e. small holes). |
|  | Poor | Poor maintained, uneven playing surface (if hard court), inadequate amounts of sand (if beach volley), substantial defects on nets. |
| Tennis courts | Number and coordinates |  |
| Condition of facility | Good | Well maintained, even playing surface, enough sand (clay court), lighting, intact nets. |
|  | Fair | Fair maintained, uneven playing surface in some areas, inadequate amounts of sand in some areas (if clay court), minor defects on nets (i.e. small holes). |
|  | Poor | Poor maintained, uneven playing surface, inadequate amounts of sand, substantial defects on nets. |
| Basketball courts | Number and coordinates |  |
| Condition | Good | Well maintained, even playing surface, lighting, intact hoops. |
|  | Fair | Fair maintained, uneven playing surface in some areas, minor defects on hoops (i.e. small holes). |
|  | Poor | Poor maintained, uneven playing surface, substantial defects on hoops. |
| **Other sports fields** | Number and coordinates |  |
| Condition | Good | Well maintained, even playing surface, lighting, intact hoops/nets/goals. |
|  | Fair | Fair maintained, uneven playing surface in some areas, minor defects on hoops/nets/goals (i.e. small holes). |
|  | Poor | Poor maintained, uneven playing surface, substantial defects on hoops/nets/goals. |
| **Skate or BMX park/bowl** | Number and coordinates |  |
| Condition | Good | Well maintained, free of cracks and rust on ramps and rails. |
|  | Fair | Fair maintained, some rust or cracks on ramps and rails |
|  | Poor | Poor maintained, substantial rust or cracks on ramps and rails. |
| **Outdoor fitness facilities** | Number and coordinates |  |
| Condition | Good | Well maintained, free of cracks and rust. |
|  | Fair | Fair maintained, can have pale colors, some rust or cracks, typically older facilities |
|  | Poor | Poor maintained, substantial rust or cracks on the facility. |
| **Aquatic facilities** | Number and coordinates |  |
| **Indoor gyms** | Number and coordinates |  |
| **Other indoor facilities** | Number and coordinates | Could be a climbing center, gymnastics hall, etc. |
